# The combined rhizoremediation by a triad: plant-microorganism-functional materials

**DOI:** 10.1007/s11356-023-28755-8

**Published:** 2023-07-21

**Authors:** Katarzyna Chojnacka, Konstantinos Moustakas, Marcin Mikulewicz

**Affiliations:** 1 Department of Advanced Material Technologies, Faculty of Chemistry, Wrocław University of Science and Technology, Smoluchowskiego 25, 50-372 Wrocław, Poland; 2 School of Chemical Engineering, National Technical University of Athens, 9 Iroon Polytechniou Str., Zographou Campus, 15780 Athens, Greece; 3 Department of Dentofacial Orthopaedics and Orthodontics, Division of Facial Abnormalities, Medical University of Wroclaw, Wroclaw, Poland

**Keywords:** Organic pollutants, Heavy metals, Rhizodegradation, Phytoremediation, Soil amendments, Microorganisms

## Abstract

The article describes new strategies for the remediation of soils contaminated with organic and inorganic pollutants. The aim of this study is to investigate the synergistic effects of combining plant-microorganism-functional materials for a more effective reduction of soil contamination with toxic chemicals. The innovative triad involves functional materials as a habitat for microorganisms, which helps to control the release of pollutants into the soil solution from the adsorbed form. This, in turn, reduces the toxic effect on microorganisms and plants. Microorganisms play a complex role, consisting of partial biodegradation of pollutants, stimulation of plant growth, and support for nutrient supply. Plants synthesize root exudates that facilitate microorganisms in biodegrading organic pollutants and stimulate their growth. The plant takes up pollutants through the root system, which can be further supported by endophytic microorganisms. The cooperation of the three players produces a synergistic effect that enhances the effectiveness of rhizodegradation supported by functional materials, which is more effective than using microorganisms, phytoremediation, or functional materials alone. The combination of physicochemical methods (functional materials) and microbiological methods (bacteria and fungi, rhizosphere, symbiotic and non-symbiotic) supported by plants (hyperaccumulators) is a promising approach for reducing chemicals from soil. Key examples of the synergistic effects of combining plant-microorganism-functional materials have been provided in this article.

## Introduction

Rhizoremediation supported with functional materials is a novel soil remediation method which combines microorganisms, plants, and functional materials to intensify the efficiency of the process. Despite the interest in different soil remediation techniques, there are no studies that have reviewed the progress of the combination of bioremediation, phytoremediation, and remediation with functional materials. Therefore, it is important to introduce the topic and underline its significance for the field. The aim of this work is to broaden our understanding of rhizoremediation with the support of functional materials. Although most studies focus on reviewing rhizoremediation itself, this paper describes the combined application with functional materials. This emphasizes the importance of synergistic effects reached by the simultaneous use of different species of rhizosphere microorganisms, special species of plants, and materials. Our study provides the foundation for a new way of thinking about novel strategies in natural soil remediation processes that are environmentally friendly and consistent with the idea of sustainable development.

The importance of plant metabolism during bioremediation processes has been increasingly recognized in recent years. Biotransformation, a process in which microorganisms and plant metabolism work synergistically to transform and metabolize toxic contaminants into less harmful forms, is a key component of bioremediation. This review discusses the factors determining bioremediation effectiveness, the importance of plant metabolism during bioremediation processes, and the ongoing research into the use of different plant species to optimize bioremediation effectiveness (Ali et al. 2013). Factors determining bioremediation effectiveness include the type and concentration of pollutants, soil properties, environmental conditions, and the specific plant species and their associated microorganisms. Several metabolic pathways, such as glycosylation, glutathione conjugation, and the formation of metal-binding proteins, are involved in the detoxification and sequestration of pollutants within plant tissues (Ma et al. 2016).

Different plant species have varying abilities to uptake and metabolize contaminants, which is why the selection of suitable plant species for rhizoremediation is essential. For example, hyperaccumulator plants, which can uptake and tolerate high levels of specific contaminants, are ideal candidates for bioremediation. Furthermore, the efficiency of bioremediation can be improved by utilizing plants with extensive root systems that facilitate the interaction between plant roots and the rhizosphere microbial community, promoting biotransformation processes (Weyens et al. 2009). Ongoing research into the use of different plant species to optimize bioremediation effectiveness has led to the identification of several promising candidates. For instance, the use of Brassica species for the bioremediation of heavy metals and Salix species for the remediation of organic pollutants has shown promising results in field studies (Ma et al. 2011).

To induce effective bioremediation processes during rhizoremediation, it is essential to monitor and manage soil pH, electrical conductivity, and cation exchange capacity. The soil pH influences the availability of nutrients and the activity of microorganisms responsible for biodegradation. It also affects the solubility and mobility of pollutants in the soil. An optimal pH range (generally between 6 and 8) is necessary to support the growth and metabolism of the microorganisms involved in rhizoremediation. Electrical conductivity is a measure of the soil’s ability to conduct electricity, which is directly related to the concentration of dissolved salts and nutrients. High electrical conductivity may indicate increased salinity, which can adversely affect plant growth and microorganism activity. Ensuring that the soil has an appropriate electrical conductivity is crucial for promoting efficient rhizoremediation processes. Cation exchange capacity is the soil’s ability to hold and exchange positively charged ions (cations) such as potassium, calcium, and magnesium. A high cation exchange capacity indicates that the soil has a greater capacity to retain and supply essential nutrients to plants and microorganisms. This directly influences the success of rhizoremediation, as it ensures that the plants and microorganisms have adequate nutrients to support their growth and metabolic activities(Kidd et al. 2015).

It is also important to consider the types and levels of contaminants present in the soil during rhizoremediation, as different types of contaminants require different remediation strategies and the levels of contamination can impact the efficiency and effectiveness of the remediation process. In addition, it is important to consider the potential for contaminant migration and leaching during the remediation process, and the use of functional materials such as activated carbon or zeolites can help to mitigate these effects. Moreover, cost-effectiveness should be taken into account, and the long-term benefits of effective soil remediation, including improved ecosystem health and reduced public health risks, should be considered in the decision-making process. By taking into account all of these factors, rhizoremediation supported with functional materials can be a promising approach for addressing the global issue of soil contamination. In addition to soil resources, other environmental factors can also affect the success of rhizoremediation. For instance, temperature, moisture, and light availability can impact the growth and activity of microorganisms and plants involved in the bioremediation process. Therefore, it is important to monitor and control these factors to ensure optimal conditions for rhizoremediation.

Another important consideration for rhizoremediation is the potential for unintended ecological consequences. For example, the introduction of non-native plant species or microorganisms can have unintended impacts on the surrounding ecosystem. Additionally, the release of transformed contaminants into the environment can have unintended consequences, such as the potential for the formation of harmful byproducts. Therefore, it is important to conduct a thorough environmental risk assessment before implementing a rhizoremediation strategy. The effective use of microorganism-assisted phytoremediation with functional materials is important for food safety (Oladoye et al. 2022). The area of rhizoremediation supported with functional materials is attracting considerable interest because of the synergy by the combined action of a triad of functional materials, bacteria, and plants. This work describes various aspects of this process that could make the implementation of this method possible. Also, important information on rhizodegradation combined with functional materials in which each player plays a specific role is described. Functional materials adsorb pollutants and release them in a controlled equilibrium way that significantly reduces the toxicity of pollutants to both microorganisms and plants. Another role is to be the habitat for the growth of rhizospheric microflora and to be the source of growth nutrients. Microorganisms (symbiotic and non-symbiotic, bacteria, and fungi) act as biofertilizers and plant growth biostimulants producing compounds that enhance the growth of plants, solubilizing nutrients that are present in non-available soil pools, and partially biodegrade pollutants. The plant produces root exudates that support the growth of microflora and the uptake of pollutants by the roots (Molina et al. 2021). Then, depending on the involved plant, it translocates the contaminant or its metabolites into tissues through a specific process, which may be supported by endophytic materials. Another issue is the management of plant biomass after the soil remediation process. In plants that bioaccumulate the pollutant, it can be a problem if the plant becomes food or feed, posing a toxic effect (McGuinness and Dowling 2009).

Rhizodegradation is a process in which pollutants are biodegraded into less toxic substances by the action of microorganisms living in the rhizosphere. In this process, plants excrete root exudates that stimulate the growth of microorganisms capable of degrading pollutants. In contrast, rhizoattenuation is a process in which pollutants are immobilized and their mobility is reduced by the interaction between the plant roots and the soil. This process relies on the ability of plants to absorb and accumulate pollutants in their tissues, effectively reducing their availability in the soil. The use of functional materials in rhizoremediation can enhance both rhizodegradation and rhizoattenuation processes, leading to more effective soil remediation. It is important to note that the effectiveness of both rhizodegradation and rhizoattenuation can vary depending on several factors such as the type of contaminant, soil characteristics, and plant-microorganism interactions (Ouvrard et al. 2014).

The prevalence of toxic chemicals is significant, with high levels of contamination found in various regions around the world. For example, heavy metals such as lead and cadmium have been found to be prevalent in industrialized regions, while persistent organic pollutants such as polycyclic aromatic hydrocarbons (PAHs) are present in many areas due to their widespread use. Pesticides and herbicides are also widely used in agricultural practices, leading to contamination of soil and groundwater. The exact prevalence of these chemicals varies by region and type of contaminant, but their negative impacts on human health and the environment are significant. The World Health Organization (WHO) estimates that around 1.3 million people die each year due to exposure to outdoor air pollution, mainly in low- and middle-income countries. Additionally, according to the United Nations Environment Programme (UNEP), there are over 140,000 synthetic chemicals registered for use worldwide, and the production and use of these chemicals continue to grow. In addition, a study conducted by the European Chemicals Agency (ECHA) found that there are over 21,000 chemicals produced or imported in quantities exceeding one tonne per year within the European Union. Among these, about 2000 substances are considered to be of high concern due to their potential for causing harm to humans and the environment. Therefore, the need for effective and sustainable remediation techniques, such as rhizoremediation supported with functional materials, is becoming increasingly important to address the global issue of soil contamination (Saravanan et al. 2020; Hoang et al. 2021a).

Soil contamination is a global environmental problem that poses a threat to human health and ecosystems. Heavy metal pollution, pesticide residues, and hydrocarbon contamination are among the most common types of soil pollution, which can persist in soil for decades and even centuries. Traditional methods for soil remediation such as physical and chemical treatments are expensive and often ineffective. Hence, the need for alternative approaches that are cost-effective, efficient, and environmentally friendly. The combination of bioremediation, phytoremediation, and functional materials presents a promising approach to soil remediation. The use of microorganisms and plants together with functional materials can enhance the remediation of contaminated soil, and the synergy between these three players can result in a more effective method than using each one separately. This study aims to broaden our understanding of the triad of microorganisms-plants-functional materials in rhizoremediation, with the hope of contributing to the development of sustainable soil remediation techniques.

Table 1 provides information on the prevalence and environmental impact of selected pollutants. Heavy metals, such as Cd and Pb, are commonly found in industrial waste, mining sites, and contaminated water sources. They can accumulate in the food chain, cause soil contamination, and affect human health. PAHs, which are often produced from incomplete combustion of organic matter, can cause soil and water contamination, and some are carcinogenic. Phenols are toxic at low concentrations and can contaminate water and soil, while dyes can cause water pollution and affect aquatic life. PhACs, which are active ingredients in medications, can contaminate water sources and disrupt aquatic ecosystems. Effective strategies for reducing the release of these pollutants into the environment are necessary to mitigate their negative impacts.

**Table 1:** Prevalence and environmental impact of selected pollutants

Pollutant	Description	Environmental impact	Prevalence
Heavy metals	Metals with high density that can be toxic to organisms and the environment even in low concentrations	Can accumulate in the food chain, cause soil contamination, and affect human health. Some heavy metals like Cd and Pb are highly toxic and can cause irreversible damage to organs and the nervous system	In industrial waste, mining sites, and contaminated water sources. (Tchounwou et al. 2012)
Polycyclic aromatic hydrocarbons (PAHs)	Organic compounds containing multiple aromatic rings, often produced from incomplete combustion of organic matter	Can cause soil and water contamination, are toxic to organisms, and some are carcinogenic. PAHs can also disrupt the endocrine system and have negative effects on human health	In urban areas, near industrial facilities, and in contaminated soils. (Maliszewska-Kordybach et al. 2008)
Phenols	Aromatic organic compounds with a hydroxyl group attached to a benzene ring	Can cause water and soil contamination, are toxic to aquatic life, and affect human health. Phenols are toxic at low concentrations and can have negative effects on the liver, kidneys, and nervous system	In industrial effluents, agricultural runoff, and municipal wastewater. (Arora et al. 2014)
Dyes	Synthetic colorants used in various industries, such as textiles, paper, and food processing	Can cause water pollution and affect aquatic life. Some dyes are toxic and carcinogenic. Reactive blue 19 is a common textile dye that can cause skin irritation and respiratory problems	In industrial effluents, especially from textile and dyeing industries. (Al-Tohamy et al. 2022)
Pharmaceutical active compounds (PhACs)	Active ingredients in medications, including antibiotics, hormones, and anti-inflammatory drugs	Can cause water contamination, contribute to antibiotic resistance, and disrupt aquatic ecosystems. PhACs can also have negative effects on human health, such as endocrine disruption and antibiotic resistance	in wastewater, surface waters, and groundwater due to human and veterinary usage. (Kümmerer 2009)

Phytoremediation and rhizoremediation are two related techniques that use plants and their associated microbes to remediate contaminated soils and water. Phytoremediation involves the use of plants to remove, stabilize, or degrade pollutants from the environment. In contrast, rhizoremediation is a specific type of phytoremediation that focuses on the interactions between plants and the microorganisms in their root zone (rhizosphere) to enhance pollutant removal.

Recent literature has highlighted the potential of both phytoremediation and rhizoremediation as effective and eco-friendly techniques for remediating contaminated sites. For example, a review by Yuliasni et al. (2023) discusses recent progress in phytoremediation-based technologies for industrial wastewater treatment (Yuliasni et al. 2023). The authors highlight the potential of various plant species, such as *Typha angustifolia* and *Phragmites australis* (Cav.) Trin. ex Steud, for removing pollutants such as heavy metals and organic compounds from wastewater.

In terms of rhizoremediation, recent studies have explored the use of microbial consortia to enhance the efficiency of pollutant removal. For example, a study by Wang et al. (2022) demonstrated the effectiveness of a microbial consortium containing *Rhodococcus qingshengii* in removing Pb, Cd, Cu, and Zn from contaminated soil (Wang et al. 2022). Other studies have investigated the use of functional materials, such as biochar, to enhance the effectiveness of rhizoremediation (Tripathi et al. 2017).

This paper will employ the suggested definition of rhizoremediation, which is a fusion of the concepts of bioremediation and phytoremediation. The article is organized as follows and has been divided into the following parts: “Introduction” introduces the idea of rhizoremediation, and “Soil pollution” discusses soil pollution. This is followed by “The role of microorganisms and plants in rhizoremediation.” The “Functional materials in rhizoremediation” section is on functional materials in rhizoremediation. The “Examples of combined plant–microbe-functional materials in the context of contaminated soil remediation” section reviews examples of combined plant-microorganism-functional materials. Finally, the paper will conclude with “Future perspectives” and “Conclusions.”

## Soil pollution

Globally, soil conditions are gradually deteriorating, resulting in negative effects on food security and human health (Rebello et al. 2021; Awange and Kyalo Kiema 2013). Soil contamination with organic (xenobiotics, persistent organic) and inorganic pollutants (toxic elements) is an obstacle to the production of healthy food (Ali et al. 2021; Press 2022). Different bioremediation strategies have been developed taking into account the source of the pollutant, its biogeochemical cycle, absorption, toxicity, and detoxification (Chen et al. 2021). On this basis, the following chemical, biological (phytoremediation, biosorption, and bioaccumulation, bioreactors, biostimulation, composting, bioleaching, bioventing), and physicochemical (landfill, acid leaching, electroreclamation, excavation, thermal treatment) methods have been elaborated (Ali et al. 2021). The problematic pollutants removed from soil include HMs (heavy metals), PCBs (polychlorinated biphenyls), PAHs (polycyclic aromatic hydrocarbons), PFRs (persistent free radicals), VOCs (volatile organic compounds), pesticides, petroleum hydrocarbons, and oils (Yang et al. 2022; Ren et al. 2018; Terzaghi et al. 2020; Uribe et al. 2022; Rostami et al. 2021; Ying et al. 2007).

The pollutants that can be removed by combined rhizoremediation include plastics, hydrocarbons, surfactants, polychlorinated biphenyls, and radioactive waste and toxic elements. Several microorganisms, including fungi and bacteria, are capable of biodegrading a wide range of pesticides, such as endosulfan, cypermethrin, pyrethroid, and profenofos, among others (Rebello et al. 2021; Saravanan et al. 2020).

### Toxic organic compounds

An effective solution in the reclamation of POPs (persistent organic pollutants) is the partnership between plants and microorganisms of a synergistic nature (Arslan et al. 2017). POPs have a high bioaccumulation factor, high toxicity, and risk to humans and organisms in the environment (Arslan et al. 2017). The bioavailability of organic pollutants in the soil is reduced by immobilization. Therefore, the adsorption to soil particles significantly reduces the bioavailability of plants. Organic pollutants adsorbed onto the soil particles depend on their physical properties and classification. If the soils contain a large part of clay, then chemicals become adsorbed. In acid soils (below pH 5.5), the degree of adsorption will be lower due to competition with protons (Saha et al. 2017). The high activity of soil microorganisms affects the biodegradation of toxic compounds. This process is reflected by the determined half-lives. It has been shown that in soils with a higher organic carbon content, half-times are shorter (Jacoby et al. 2017). Thus, such soils favor the biodegradation of organic pollutants. The introduction of additives (such as humic acid) into soils has been shown to increase the rate and efficiency of biodegradation of organic compounds (Cao et al. 2022).

### Toxic elements

Toxic elements are especially dangerous because they are not biodegradable. They accumulate in the environment because they are persistent. Contamination with toxic metals causes the degradation of soils (Ali et al. 2021). The following elements are listed among the group of toxic elements: Cd, Pb, Hg, and As. So far, their beneficial role in living organisms has not been demonstrated. Industrial activity and continuous emission can lead to very high levels of toxic elements in soil (Tan et al. 2020). Toxic metals originate from diversified anthropogenic activities. Soil contamination can be local (industrial origin) or dispersed (agricultural source) (McGuinness and Dowling 2009). The sources of these pollutants are production plants, urbanized areas, and metallurgy/mining (Ali et al. 2021). Their behavior in soil depends on pH and ionic properties. Toxic metal ions in the soil become co-precipitated with inorganic ions (phosphates, carbonates) and undergo cation exchange and chelation with organic matter (humic and fulvic acids) or with minerals (clay, oxides) (Chien et al. 2018). Metal ions can be removed from soil by the combined activity of microflora and plants: rhizoremediation, rhizofiltration, phytostabilization, and phytoextraction (Kushwaha et al. 2018). The phenomenon of biomineralization of metals by plants is also being indicated. It is a defense system against the toxicity of metals (Adele et al. 2021).

**Fig. 1:**
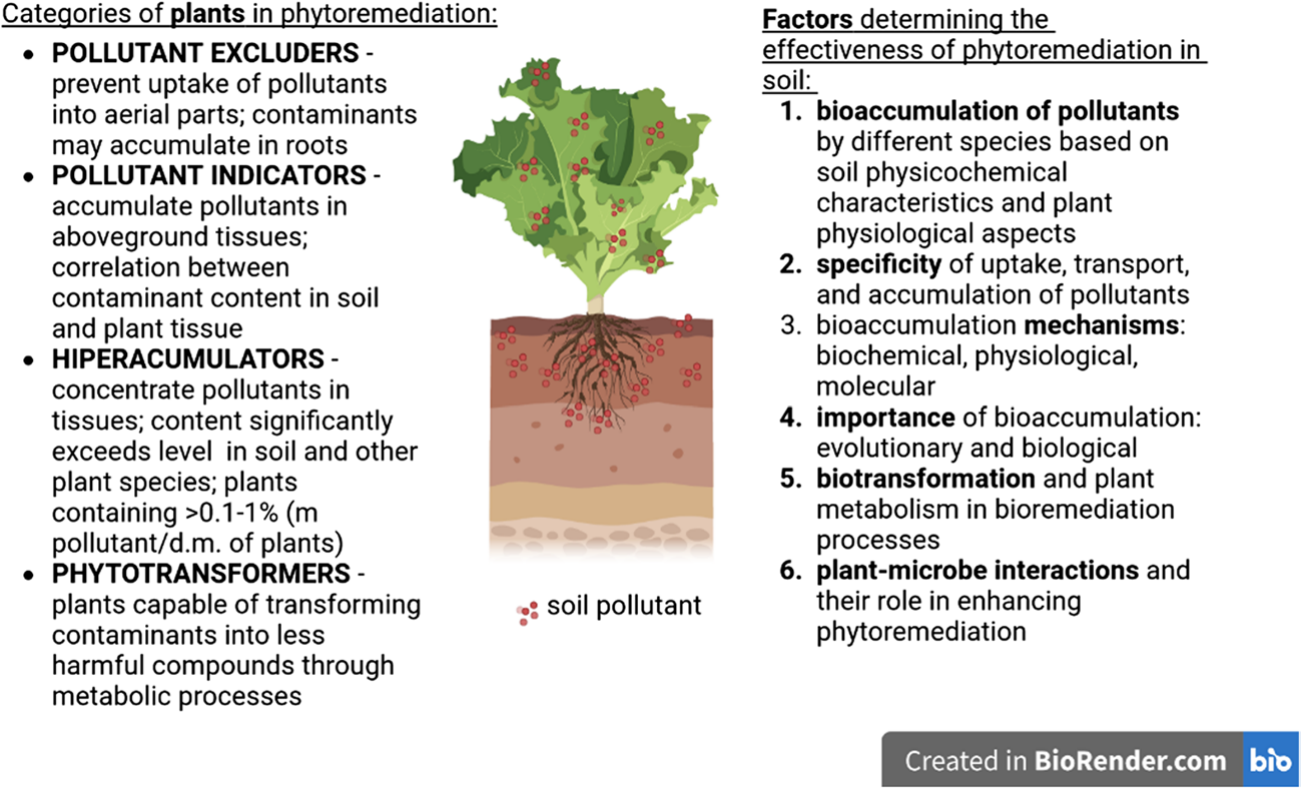
Key elements of phytoremediation categories and factors determining its effectiveness in soil (Memon and Schröder 2009) (Milić et al. 2021)

In bioremediation, toxic metal ions can be transformed into an organic complex or other ionic forms. Microorganisms synthesize siderophores which facilitate the extraction of metal ions from soils (Kushwaha et al. 2018). In contaminated areas, certain plant species can grow. These plants have developed various types of protective mechanisms that reduce the uptake of pollutants from soils and those that tolerate high tissue contents (bioaccumulation) due to the developed detoxification and accumulation mechanisms. Some plants take up and accumulate toxic elements that do not perform useful biological functions: Hg, Pb, Cd, and Ag. These plants can be used in soil phytoremediation applications (Memon and Schröder 2009). Figure 1 summarizes the key elements of phytoremediation, including the categories of plants involved (excluders, indicators, and hyperaccumulators) and the factors that determine the effectiveness of phytoremediation in soil (bioaccumulation, specificity, mechanisms, and importance).

### Radioactive elements

For toxic radionuclides derived from nuclear waste, the following tests shall be carried out: source identification, biogeochemical processes, phytouptake, and phytotoxicity tests (Chen et al. 2021). Radionuclides do not play a beneficial role in living organisms (Ding et al. 2019). Their toxic effects rely on germination inhibition, plant growth, photosynthesis, nutrient absorption interference, genetic toxicity, and oxidative damage (Chen et al. 2021).

Radionuclides are present in soil and water due to various anthropogenic and natural sources, and their levels can vary greatly depending on the location and type of source. The concentration of radionuclides is usually measured in units of becquerels (Bq) per gram or microgram of soil or water. For toxic radionuclides derived from nuclear waste, various tests should be carried out, including source identification, biogeochemical processes, phytouptake, and phytotoxicity tests, to determine their effects on the ecosystem (Chen et al. 2021). Radionuclides do not play a beneficial role in living organisms and can have various toxic effects, including germination inhibition, plant growth, photosynthesis, nutrient absorption interference, genetic toxicity, and oxidative damage (Chen et al. 2021; Ding et al. 2019). The severity of these effects depends on the concentration and duration of exposure to radionuclides. Therefore, it is crucial to monitor and manage the levels of radioactive contaminants during rhizoremediation processes to ensure that they are below the safe limits set by regulatory authorities. This can be achieved by implementing appropriate remediation strategies such as the use of hyperaccumulator plants or functional materials like zeolites or activated carbon. By considering the concentration of radionuclides and other factors that determine bioremediation effectiveness, rhizoremediation can be an effective approach to remediate radioactive contamination in soil and water.

### Plastics and microplastics

Until now, a single-use plastic of 8.3 billion tons has been manufactured. The toxic effects of plastics on soil microorganisms and other soil organisms have been demonstrated. (Amobonye et al. 2021). The recently signaled problem is the occurrence of microplastics (MPs), also in the soil environment (Lares et al. 2019). These are plastic particles of size less than 5 mm that easily migrate across the trophic chain to which humans are exposed. Microplastics are being bioaccumulated from the soil environment (Amobonye et al. 2021). MPs in soil cause bioaccumulation and toxic effects in plants and animals. Recent research has shown that microorganisms play a role in the degradation of these persistent pollutants (Kaur et al. 2022). Bacterial and fungal species that have the ability to biodegrade MPs were identified (Dos Santos et al. 2023). These are, for example, *Rhodococcus* sp. or *Bacillus* sp. Bacteria and plastic-degrading fungi have also been isolated from the root zone of plants (Venkatesh et al. 2021). This microflora is also present in the intestines of worms (*Tenebrio molitor* L.) and in fungi (*Aspergillus flavus* Link), isolated from the intestinal tracts of wax moths (KAUR et al. 2022). It was found that natural microflora had little capacity to degrade MPs (CF et al. 2021). Research on genetic modifications and the isolation of enzymes supporting this process is underway (Kaur et al. 2022). In order to make progress in this area, it is necessary to identify the metabolic pathways for the degradation of various types of microplastics (Miri et al. 2022). Inspiration may also be provided by microorganisms used in the degradation of microplastics (Kaur et al. 2022).

## The role of microorganisms and plants in rhizoremediation

The effective use of plant-assisted rhizoremediation is important for food safety (Oladoye et al. 2022). Key factors to select a plant for phytoremediation are as follows: plant growth rate, root/rhizosphere characteristics (characteristics, architecture, physicochemical properties), adaptability, environmental tolerance and response, contamination (bioavailability and toxicity), and soil (type) (Oladoye et al. 2022). Beneficial interactions in rhizoremediation between microorganisms and plants consist of the combination of plant action (phytoextraction/degradation/stabilization, synthesis of plant secondary metabolites) and microbial activity (biofertilization, reduction of pollutants bioavailability, stem biostimulation, biocontrol) (Vergani et al. 2017). The role of metabolites synthesized by plants is as follows: biosurfactants (rhamnolipids, surfactin, and trehalose lipids) cause a greater desorption of hydrophobic pollutants (e.g., naphthalene, PCBs), because of which these compounds are more bioavailable for microorganisms (Vergani et al. 2017).

From contaminated soil, due to microbial action, it is possible to obtain the simultaneous effect of improving soil fertility and soil bioremediation, thus achieving the effect of healthy and productive soil (Rebello et al. 2021). The role of soil microorganisms also increases the bioavailability of nutrients and synthesis of the plant growth regulators. At the same time, they participate in various transformations of organic matter in the soil and lead to the degradation of xenobiotics. An additional function is to participate in the adsorption and desorption of various chemicals and detoxification of organic and inorganic pollutants (Rebello et al. 2021). There are strains that can break down specific pollutants (Rebello et al. 2021). Conventional bioremediation techniques include biostimulation (adding nutrients to stimulate microorganism activity) or bioaugmentation (introducing external microflora) (Ren et al. 2018).

Many studies compare phytoremediation, bioremediation, and a combination of these two techniques. The conclusion clearly indicates the synergistic effect of the plant–microbe partnership (Chen et al. 2021). Plants produce substances that support the colonization of the root zone by microorganisms through progression, adsorption, and by polymers. This symbiotic arrangement helps the plant to overcome biotic and abiotic stress (Mitra et al. 2021). The role of plants is to provide a habitat for bacteria and also for nutrients. The function of microorganisms is to support plant growth and health. Active microorganisms are primarily rhizobacteria that grow near the roots and sometimes develop in plant tissues without being pathogenic (endophytic) (Arslan et al. 2017). Microorganisms use contaminants as a growth substrate. They support the plant in the reduction of the stress caused by the presence of contaminants. These bacteria stimulate root and shoot growth and thus increase the habitat available to microorganisms (Arslan et al. 2017). Bacteria possess catabolic enzymes encoded in specific genes that are used to metabolize and therefore detoxify persistent organic pollutants (POPs) (Arslan et al. 2017). Phytouptake includes translocation, followed by phytodegradation or phytovolatilization. However, the degradation processes involving functional materials such as titanium nanodioxide, silicon nanoparticles, or carbon nanotubes are poorly described, and the specific roles of these materials are not explained. Rood exudates, plant growth–promoting substances (PGP), nutrients, and water (sequestration, degradation, phytohormones) support rhizobacteria in transformation, enzymatic degradation, and mineralization to H_2_O, CO_2_, and Cl_2_. The key issue is the bioavailability of soil plants and soil conditions (organic material, redox potential, microorganisms, soil pH) affecting soil–plant pollution transfer. In some plants, bioaccumulation occurs mainly at the root (with limited translocation). In others: translocation to the aerial parts (Chen et al. 2021). Plant defense strategies include the synthesis of antioxidant enzymes, phytochelatins, and compartmentalization. In the process of plant remediation, soil organic matter (SOM) is an important factor that influences its efficacy. Other factors include plant species, bioavailability, environmental conditions (pH, temperature, soil oxygenation), and aging time (Gabriele et al. 2021). This is summarized in Table 2. The characteristics of various strategies for the bioremediation of toxic metals are shown in Table 3.

**Table 2:** The mechanism of cooperation of microorganisms with the plants (Mitra et al. 2021; Rengel and Marschner 2005; Henagamage et al. 2022)

Microorganism		Metabolites	Role
PGPR	Plant growth–promoting rhizobacteria	- Synthesize metabolites: - HM chelating agents (metallophores, organic acids, biosurfactants, siderophores), - Phytohormones (IAA, CK, GA) - Enzymes (ACC deaminase, 1-aminocyclopropane-1-carboxylic acid)	Affect the availability of nutrients (macro- and micro-) and pollutants, protect against stress
AMF	Arbuscular mycorrhizal fungi		Stimulate plant growth, protect against stress related to the presence of pollutants
EB	Endophytic bacteria		Penetration into the root tissue and other tissues of the plant

*CK* cytokinins, *HM* heavy metals, *GA* gibberellic acid, *IAA* indole-3-acetic acid

**Table 3:** Strategies of bioremediation of toxic metals (Kushwaha et al. 2018; Bilal et al. 2020; Gabriele et al. 2021; Milić et al. 2021; Kushwaha et al. 2018; Alori et al. 2022; Hidangmayum et al. 2022; Al-Tohamy et al. 2022; Henagamage et al. 2022; Kafle et al. 2022)

Method	Characteristics
1. Phytoremediation	Plants can reduce mobility, toxicity, and the content of pollutants The activity of the microorganisms in the root zone is stimulated The rhizosphere is a place of biological, physical, and chemical interaction Root exudates are released by plant that stimulate microbial growth and uptake
1.1 Phytostabilization	The accumulation in the soil and leaching into the groundwater are reduced Metal-tolerant plant species Metal-binding soil amendments that increase pH (lime) and organic matter content that reduce bioavailability The absorption and accumulation of plant roots immobilize toxic pollutants and prevent leaching into groundwater Plants that highly bioconcentrate pollutants and effectively translocate them are selected
1.2 Phytoextraction	Uptake by plant roots, translocation, and accumulation in plant tissues The plants are harvested and incinerated Selection of plants: hyperaccumulators that tolerate pollutants (no symptoms of toxicity), quick growth, high biomass, and large roots The formation of stable metal ions complexes is supported by the addition of organic acids (citric, malic) and chelators (DTPA, HEDTA, CDTA, EDDS, EDDHA)
1.3 Rhizofiltration	Plants accumulate, concentrate, and precipitate contaminants in the root system that are facilitated by the excretion of root exudates. Roots/plants are harvested and removed
2. Immobilization by bacteria	Microorganisms reduce uptake by plants by biosorption or bioaccumulation processes that are supported by sequestration of specific metabolites, chelation, biotransformation, detoxification, and precipitation
2.1 Biosorption/complexation	Bacteria biosorb contaminants to functional groups located on their surfaces: carboxyl, hydroxyl, amino, phosphate, or take up within cells (bioaccumulation). Microorganisms activate the synthesis of proteins that bind to toxic metal ions. Also, polymers are synthesized and released extracellularly
2.2 Precipitation	Microorganisms activate precipitation of insoluble metal compounds (carbonates, phosphates, hydroxides, and sulfur compounds) by producing specific ions extracellularly or precipitating on the cell wall

*CDTA* trans-1,2-diaminocyclohexane

*N,N,N′,N′* tetraacetic acid

*DTPA* diethylenetriaminepentaacetic acid

*EDDHA* ethylenediamine-di-o-hydroxyphenylacetic acid

*EDDS* SS-ethylene-diamine disuccinic acid

*HEDTA* N-hydroxyethylenediaminetriacetic acid

Rhizoremediation is the combination of bioremediation and phytoremediation—pollutants are bound by plant roots. Plant roots secrete saponins and tannins. These are compounds that chelate toxic metal ions from the soil solution (Ali et al. 2021). Rhizosphere is the soil surrounding the root environment that contains microorganisms. Roots secrete certain metabolites that support the activity of microorganisms in the root zone. Identification of the phenomena that occur in the rhizosphere is crucial for biodegradation and bioaccumulation of pollutants in soil environments (Singh et al. 2017).

By changing chemical speciation, metal toxicity and bioavailability can be modified (Guo et al. 2021). Additionally, there are different gradients within the rhizosphere: pO_2_, pCO_2_, redox potential, pH, metal ions, concentration, and concentration of organic ligands (Adele et al. 2021). Organic substances released in the root area were also shown to change soil solution pH and metal ion solubility (Adele et al. 2021). One of the mechanisms of phytoremediation combined with functional material process is phytoextraction. When organic compounds are removed (e.g., pyrene), the effectiveness of the process is increased by the introduction of soil surfactants and chelating agents (Kafle et al. 2022). In this way, the accumulation of pyrene has been shown to be higher by 20%. Microbial inoculation can increase bioaccumulation by 30–40% due to the presence of pyrene-degrading bacteria in the root zone. The introduction of fungal inoculants also has a beneficial effect (Rambal 2021; Ani et al. 2021). Another efficiency-increasing technique is the introduction of additives such as biochar or non-ionic surfactants (Gabriele et al. 2021; Li et al. 2020).

### Endophytic bacteria

Endophytic bacteria (bacteria naturally present in plants that are not pathogenic) and rhizospheric (root colonization) support phytoremediation (McGuinness and Dowling 2009). Endophytic bacteria play a role in improving the expression of catabolic genes and controlling the catabolic pathways of bacteria (McGuinness and Dowling 2009). Several studies have recently described the beneficial role of endophytic bacteria in phytoremediation from organic compounds and toxic elements. Endophytic bacteria reduce toxicity (decontaminate, e.g., toxic elements) by activating their own catabolic pathway. In this way, they provide the plant with protection against the stress caused by the presence of contaminants in soil (Ma et al. 2016). The mechanism of action of endophytic bacteria is by direct action consisting of the production of substances that promote plant growth (enzymes, phytohormones, siderophores, substances solubilizing nutrients (NPK) and indirectly by protecting against biotic stress (pathogens) or supporting plant immunity) (Wu et al. 2022). Endophytic bacteria also secrete extracellularly (into the plant organism) biosurfactants, and polymers that, for example, immobilize toxic elements, thus excluding them from metabolism (Arslan et al. 2017). The activity of these bacteria has an impact on phytoaccumulation, translocation, and detoxification of pollutants (Ma et al. 2016).

### “Superbugs”

Additionally, there exist microorganisms known as “superbugs” that possess the ability to biodegrade a wide range of contaminants. Consortia of microorganisms, in which individual strains perform complementary functions, also have useful properties. For example, a consortium of *Pseudomonas* sp., *Enterobacter* sp., *Aspergillus* sp., and *Rhodotorula* sp. can biodegrade as many as seven different pesticides (Rebello et al. 2021). The consortium of microorganisms is created by various methods, e.g., stimulating the growth of microorganisms by introducing nutrients, and cultivation under conditions of optimal temperature and humidity (Singh et al. 2014).

### Transgenic organisms: plants and microorganisms

GMOs (genetically modified organisms) can conduct the biodegradation process faster because they have a modified metabolism, which enables the secretion of substances that support the biodegradation process (Rebello et al. 2021). Improvements in phytoremediation methods may include the development of new transgenic bacterial strains with increased ability to bind pollutants and produce, e.g., chelating/adsorbing proteins, producing siderophores, exopolysaccharides, and metallothionein (Mitra et al. 2021). Transgenic organisms (plants and microorganisms) are a promising (although controversial) direction from the viewpoint of soil biodegradation and detoxification itself (Mitra et al. 2021). An additional problem is the relatively low survival of these organisms in the environment, which is favored by factors that limit the effectiveness of the bioremediation process: soil nutrients, temperature, and the presence of indigenous microorganisms competing for survival, soil chemical composition, especially the content of nutrients, C:N:P ratio, pH, oxygen availability (Rebello et al. 2021; Ren et al. 2018). To understand plant and transgenic microorganism metabolism, research is needed in order to prevent it from spreading in the environment (Ojuederie and Babalola 2017).

An important direction is such a modification of the microorganism-plant system in order to strengthen the mechanisms to achieve better efficiency. In phytoremediation, it is important to protect plants against oxidative stress (Rebello et al. 2021). Therefore, measures should be taken to increase antioxidant activity to intensify the uptake of pollutants, their translocation, and sequestration (Oladoye et al. 2022).

The shortcoming presented in the literature is the modification of the genome of the plant itself to increase the phytoremediation potential. The literature primarily provides screening of various plant species for the remediation of a specific (Rebello et al. 2021).

### Metabolomics

#### Importance of omics technologies in phytoremediation

Phytoremediation has many advantages, such as low cost, low environmental impact, and aesthetic benefits. However, individual plant species used in phytoremediation have their disadvantages, including low biomass productivity, slow growth rate, and low tolerance to contamination. Therefore, there is a need for continued research and development of phytoremediation technologies, including the use of “omics” technologies such as metabolomics, proteomics, and genomics (Oladoye et al. 2022).

Metabolomics, in particular, has the potential to improve the effectiveness of phytoremediation by providing insights into the metabolic pathways involved in contaminant degradation and plant–microbe interactions. In practical applications, the use of various techniques should be combined to obtain a synergistic effect, such as combining with bacterial bioremediation, the use of composts, bacteria or plant growth stimulants, and phytohormones (Vergani et al. 2017).

The root exudates produced by plants that support rhizoremediation have not been fully identified and characterized. The mechanism of this process is still unknown, but the use of molecular methods such as metagenomics and other “omics” technologies can aid in investigating symbiotic microorganisms and the identification of enzymes participating in the rhizoremediation of toxic chemicals. Therefore, continued research into omics technologies is essential for improving the effectiveness of phytoremediation and addressing the challenges and methodological gaps in soil remediation phytotechnology.

## Functional materials in rhizoremediation

In many cases, the use of phytoremediation is described as a slow and not efficient process. This can be changed by the combined application of microbiological and physicochemical methods (Sarma et al. 2019; Alori et al. 2022). A combination of phytoremediation and functional materials such as carbon materials and nanomaterials can be employed (Gong et al. 2021; Hidangmayum et al. 2022).

Heavy metal bioavailability has been shown to affect the biodegradation of toxic organic compounds by microorganisms (Hidalgo et al. 2022). This is called the co-pollution effect. The mechanism is that toxic elements inhibit catabolic enzyme activity (Hoang et al. 2021b, a) into account contamination with several pollutants; additives such as metal ion chelating agents or sorbents and the addition of bacteria bioaccumulating toxic metals should be used (Olaniran et al. 2013).

New functional materials were used for combined remediation with carbon nanotubes and starch-stabilized nanozero-valent iron and biochar for cadmium remediation by changing the structure of microbial communities using the *Boehmeria nivea* (L.) Gaudich phytoremediation method. In this way, the phytoremediation from Cd was obtained by stimulating plant growth and increasing the degree of bioaccumulation of Cd by approximately 50%. The mechanism was shown to increase the bioavailability of Cd by transformation of the oxidizable Cd into reducible. Microbial community studies using the 16S rRNA gene sequence method were carried out. Microorganisms of the genus *Actinobacteria* sp., *Nitrospirae* sp., and *Firmicutes* sp. stimulate plant growth, reduce the toxicity of cadmium, and increase its bioavailability (Gong et al. 2021). The advantage of these methods is low investment costs (Lee et al. 2021; Yaashikaa et al. 2022). Titanium nanodioxide caused increased bioaccumulation of heavy metals in soybeans (Gong et al. 2021). Silicon nanoparticles supported phytoremediation with Cr by stimulating plant growth and uptake of toxic metals and reducing oxidative stress (Gong et al. 2021). There are also different types of biochars used: PBC (unprocessed biochar), MBC (metallic/metallic composites-biochar composite), CMBC (minerals-biochar composite), and CBC (carbon-derived materials-biochar composite) (Lin et al. 2022). The impact of biochar on the bioavailability of organic toxic compounds in soil was investigated (Gabriele et al. 2021). Biochar introduced into the soil will affect the microbial communities, plants, and fauna present there (Cao et al. 2022). The bioavailability of individual contaminants also changes (Li et al. 2019). Beneficial effects of materials obtained from biomass by pyrolysis or hydrothermal processes on rhizoremediation of organic pollutants include adsorption of organic pollutants (pesticides, antibiotics), providing nutrients and habitat for the growth of microflora, and reducing bioavailability of pollutants (Zhao et al. 2022). Negative effects resulting from intentional and nonintentional applications: adsorption of signal molecules (flavonoids, quinolones, indoles), which weakens the interactions between microorganisms, reduces their effectiveness, and inhibits gene expression, introduction of toxic compounds (VOCs, PAHs (anthracene, benzopyrene), HMs, PFRs, xylenols, acrolein, formaldehyde, dioxins), causing weight loss (Sarma et al. 2019). Positive effects include the adsorption of organic pollutants (pesticides, antibiotics) that decrease the bioavailability of pollutants. Chars also provide nutrients and habitat for the growth of microflora (Zhao et al. 2022).

## Examples of combined plant–microbe-functional materials in the context of contaminated soil remediation

An overview of studies that use rhizoremediation supported with soil amendments is presented in Table 4. Various functional materials, such as titanium nanodioxide, silicon nanoparticles, and carbon nanotubes, have been found to enhance the effectiveness of rhizoremediation. For example, titanium nanodioxide has been shown to improve the degradation of organic pollutants by promoting the formation of reactive oxygen species (ROS), which can break down contaminants. Silicon nanoparticles have been found to enhance plant growth and improve the uptake and degradation of contaminants in soil. Carbon nanotubes have also been shown to enhance the degradation of contaminants by providing a surface for microbial attachment and by improving the transport of contaminants to the rhizosphere.

**Table 4:** An overview of combined rhizoremediation plant-microorganism-functional materials

Ref.	Plant	Microorganism	Functional material/substance	Pollutant	Results	Conclusions
(Wu et al. 2022)	*Non-hyperaccumulating S. alfredii hyperaccumulating Sedum (S. alfredii and S. plumbizincicola)*	Proteobacteria, actinobacteria, acidobacteria	–	Cd, Zn	Taxa related to the hyperaccumulation of Zn and Cd are similar to each other	Nine types of bacteria showed a positive correlation with Cd/Zn hyperaccumulation
(Wang et al. 2022)	*L. perenne, V. zizanioides, B. juncea, S. alfredii, S. nigrum*	*R. qingshengii*	Catabolizing abscisic acid (ABA)	Cd, Zn, Pb, Cu	The level of Pb, Cd, Cu, and Zn increased by 28.8–331.3%, 8.5–393.4%, 21.2–222.5%, 14.7–115.5%, and 28.3–174.2%	Significant correlation of the influence of microorganisms on the concentration of Zn and Cd in plants with ABA
(Yu et al. 2022)	*B. pilosa, X. strumarium*	*Actinobacteriota* sp.*, Chloroflexi* sp.*, Acidobacteriota* sp.*, Ascomycota* sp.*, Basidiomycota* sp.*, Mortierellomycota* sp.	–	Pb, Cd, Zn	Decrease in the level of Cd, Pb, and Zn (DTPA acid)	The plants showed better ability to phytoextract metals, communities of bacteria, and fungi which may have a positive effect on the phytoextractive capacity of (hyper) accumulators
(Kushwaha et al. 2022)	*Arabidopsis halleri* (L.) O’Kane & Al-Shehbaz	Microbial consortia in rhizosphere of 4 plants		Zn, Cd	A strong relationship of Zn hyperaccumulation was observed for the variety of *A. halleri* rhizosphere fungi, while Cd hyperaccumulation was more strongly associated with the bioavailability of Cd and Zn soil	Strong relationships were observed between the properties of the rhizosphere microbial consortium and the hyperaccumulation of trace metal and metalloids by *A. halleri*
(Bhatt et al. 2022)	*Zea mays* L	*Bacillus thuringiensis* strain SG4, *Bacillus sp.* strain SG2		Cypermethrin	In vitro and pot experiments; cypermethrin bacterial degradation cypermethrin (80% and 85%) with externally supplied N	The addition of bacteria increased the degradation of cypermethrin in the soil
(Zhang et al. 2022)	*A. wardii*	–	Nitrilotriacetic acid (NTA)	Pb	Pb accumulation increased (14.3%)	The application of NTA may stimulate some microorganisms to maintain rhizosphere activity
(Ali et al. 2021)	*Symphytum officinale* L	*Streptomyces pactum* (Act12), consortium of *Bacillus* (B. subtilis and licheniformis; 1:1	–	Zn, Cd	- Zn, Cd concentration significantly decreased in shoots,—Root concentration was higher than shoots	On soils contaminated with toxic metals, *S. officinale* can be used for rhizoremediation
(Viji et al. 2021)	*Pennisetum purpureum Schumach*	VITATJM1, VITATJM2, VITATJM3, VITATJM4		Cd	VITATJM3 and VITATJM4 proved to be successful in the synthesis of HCN, IAA, NH3, and P dissolution	May be effective in removing cadmium from soil
(Akkaya 2020)	*Nicotiana tabacum* L	*P. putida* KT2440 or KT.DNT		2,4-DNT	Remediation efficiency 93–98% by *P. putida* KT.DNT	The use of 2,4-DNT-remediating bacterium that inoculated with *N. tabacum* was found efficient
(Li et al. 2019)	Soil collected		Biochar	Polycyclic aromatic hydrocarbons (PAHs)	Intensification of dispersion of PAHs by biochar, increase in soil microflora	The supplementation of biochar and rhizosphere is an efficient strategy for remediating contaminated soil
(Tripathi et al. 2017)	*Cicer arietinum* L	*Trichoderma*: - M-35 (tolerant) - PPLF-28 (sensitive)		As	Differences in the concentration of inorganic and organic arsenic compounds have been found, even though the As concentration in chickpea tissue was comparable for both *Trichoderma* treatments	Based on the study, it was found that the use of *Trichoderma* M-35 in fields with an increased As content may yield promising results
(Ely and Smets 2017)	One monocot and two dicotyledon plants	Rhizobacteria		Polycyclic aromatic hydrocarbons (PAHs)	In growth on chrysene and anthracene plates with PAHs, root zone isolates degraded PAHs of 3- and 4-ring	The phenolic compounds are associated with biodegradation of PAHs
(Gartler et al. 2014)	Different leguminous and grass species; *Medicago sativa* L		Rapeseed oil	Polycyclic aromatic hydrocarbons (PAHs)	Effect of vegetable oil on development of plant (reduction plant mass) A reduction in PAH level in soil that was not enriched with oil. In the oil-enriched soil, no decrease in PAH content was observed	No intensification of PAH decomposition by plants in soil mass. PAH content in the alfalfa rhizosphere decreased significantly
(Dadrasnia and Agamuthu 2013)	*D. reflexa*, *P. polystachyus*		Tea leaf, potato skin, soy cake	1 and 2.5% petroleum derivative–contaminated soil	The reduction of 90–99% of oil in soil polluted with 1 and 2.5% and 1% oil, by addition of soy cake, 52–62% was achieved in soil with *D. reflexa* and 84% and 91% oil reduction for *P. polystachyus*	Positive relationship for diesel remediation and plant mass. *D. reflexa* addition of organic material is more perspective for hydrocarbon removal
(Mwegoha et al. 2007)	*Salix babylonica* L		Dissolved organic carbon (DOC)	Perchlorate	Soil and hydroponic bioreactors; a) *S. babylonica* and DOC b) *S. babylonica* and no DOC and non-planted bioreactors. Both planted and unplanted soil bioreactors without DOC removed > 95% perchlorate in 8 days	The experiment carried out proved that DOC is a factor limiting the degradation of perchlorate
(Dzantor and Woolston 2001)	*Lathyrus sylvestris* L., *Phalaris arundinacea* L., and *Medicago polymorpha* L	Orange peels, ground pine needles (soil amended additionally with biphenyl)	Aroclor 1248 (PCB)	[Specify the initial concentration]	After 100 days of the experiment, PCB recovery was 69% for non-planted soils, and 65%, 59%, and 55% for the soils planted with flat peas, reed canarygrass, and burr medic, respectively. Soil modification and planting increased PCB dispersion in the soil as related to planting alone (with the exception of biphenyl and Burr medic). In soil amended with orange peel and contaminated with biphenyl, the concentration of microorganisms was higher	*Lathyrus sylvestris, Phalaris arundinacea, and Medicago polymorpha*
(Zheng et al. 2022)	*Phragmites australis* (Cav.) Trin. ex Steud	Achromobacter sp. and Bacillus sp.	Montmorillonite nanocomposite	Reactive blue 19 dye	Enhanced dye removal	Montmorillonite nanocomposite supported plant–microbe interactions and improved dye removal
(Liu et al. 2018)	*Phragmites australis* (Cav.) Trin. ex Steud	Rhizobacteria (Azospirillum spp.)	Nanoscale zero-valent iron	Hexavalent chromium (Cr(VI))	Enhanced Cr(VI) reduction and removal	Nanoscale zero-valent iron supported plant–microbe interactions and improved Cr(VI) reduction
(Huang et al. 2007)	*Juncus effusus* L	Arbuscular mycorrhizal fungi	Biochar	Heavy metals (Cd, Pb, Zn)	Enhanced plant growth and metal uptake	Biochar improved plant–microbe interactions and metal uptake in phytoremediation of contaminated soil
(Gkorezis et al. 2016)	*Zea mays* L	–	Chitosan	Cadmium	73% pollutant removal	Chitosan enhanced rhizoremediation by promoting plant growth and Cd uptake by maize
(Płociniczak et al. 2013)	*Sinapis alba* Moench. L	Enterobacter intermedius MH8b	–	Heavy metals (Cd, Pb, Zn)	Improved plant growth and heavy metal uptake	Plant–microbe interactions led to enhanced heavy metal uptake by Sinapis alba

### Petroleum derivatives

In the experimental work, reed fescue was evaluated in terms of rhizoremediation dynamics, methane emission, and the dynamics of microbial consortia in diesel-oil-contaminated soil (Hoang et al. 2021b; Kim and Cho 2022). In the results, the authors reported that the elimination of diesel fuel from the soil was 30.2% for the soil with fescue, 19.4% for the unfilled soil, and the addition of compost increased the elimination to 39.2%. The effect of concentration on CH_4_ emission was observed: higher oil concentration caused higher emission (approximately 3.8 times). The positive association of *Rhizobium* sp., *Halothiobacillus* sp., and *Geobacter* sp. species with the treatment of contaminated soil (Lee et al. 2021).

The investigators evaluated *Dracaena reflexa* Lam and *Podocarpus polystachyus* R.Br. ex Endl. in terms of their ability to remediate soils contaminated with 1% and 2.5% diesel oil. Tea leaves, soy cake, and potato peel were added to the soil. The experiment lasted 270 days. The study shows that the oil is degraded most quickly after adding soy cake (especially for *D. reflexa*). There is a positive correlation between hydrocarbon degradation and plant biomass production. It was emphasized that *D. reflexa* has a greater potential for the degradation of hydrocarbons (Dadrasnia and Agamuthu 2013).

### Insecticide: cypermethrin

Often used in agriculture and at home is cypermethrin, which is a persistent toxic insecticide that is relatively easy to transfer to soil and aquatic environments. In an experimental study, the decomposition of cypermethrin was assessed by microbial strains (*Bacillus thuringiensis* strain SG4 and SG2). The findings demonstrated that the strains broke down cypermethrin when external nitrogen sources were present, and the addition of organic materials to the soil, including fresh cow manure, gypsum mud compost, and cereal straw, enhanced the degradation process of cypermethrin. Cereal straw showed the highest impact on decomposition with half-life of the substance, 4.4 days. Pot studies with *Zea mays* L. revealed that bacterial strains enhance plant biomass growth and cypermethrin degradation (Bhatt et al. 2022).

### 2,4-dninitrotoluene

In an experiment describing the degradation of 2,4-dinitrotoluene (2,4-DNT), two bacterial strains were described: *Pseudomonas putida* KT2440 and strain KT.DNT inoculated the plant *Nicotiana tabacum* L. *(N. tabacum)*. The plant biomass was three times higher, in comparison with the control group. In the case of KT.DNT inoculation, a maximum degradation rate of 2,8-DNT of 93–98% was observed. The conclusions highlight the benefits of using *N. tabacum* plants in the remediation of soil contaminated with 2,4-dinitrotoluene (Akkaya 2020).

### PAHs

The study assessed the rhizoremediation potential of the *Novosphingobium* sp. strain. HR1 together with clover for the degradation of PAHs. The results highlight the synergy between clover and *Novosphingobium* sp. HR1a results from the use of various sources of carbon and nitrogen by bacteria, which are excreted during germination and the ability of root exudates to stimulate the degradation pathway (Molina et al.).

In another work on the remediation from PAHs, the authors verified whether the addition of rapeseed oil may pose an increased risk of degradation. Various species of legumes and grasses were studied. In the results, the authors found that the supplementation with oil from rapeseed oil resulted in the reduction of the height of plants and their biomass—the addition of oil by 1 and 3%. During the experiment, no decrease in PAHs was observed in the oil-enriched soil, while in the soil without oil, the PAH content decreased. The results highlighted a significant reduction of these pollutants in PAHs in the alfalfa root zone (Gartler et al. 2014).

The experimental work evaluated chemical relations between plants and microorganisms in the rhizosphere, which helps to decontaminate soil polluted with PAH. Isolated bacteria from the rhizosphere of monocotyledonous plants and dicotyledonous plants grown on PAH soil were subjected to laboratory tests. Polycyclic aromatic hydrocarbon (PAH) degrading bacteria have been chosen for cultivation on chrysene and anthracene. The authors found that the rhizosphere microorganisms degraded 3- and 4-ring PAHs. Flavonoids and simple phenols were found to be substrates for rhizobacteria (Ely and Smets 2017).

It has been proven in metabolic studies and sequencing that the use of biochar enhances the effect of rhizoremediation by supporting the growth and diversity of microflora, increasing the bioavailability of pollutants and inducing synthesis of metabolites by microorganisms and the plant in the rhizosphere, by which a synergistic effect is obtained (Li et al. 2019). The addition of biochar to soil was studied in the process of rhizoremediation of polycyclic aromatic hydrocarbons (PAH), taking into account metabolomics by a ryegrass plant. Biochar and rhizosphere microorganisms were shown to have a beneficial effect on degradation. The synergistic effect of the biochar root-plant microflora has been proven to promote the carbon cycle. Supplementation of biochar increased the survival of the microbiome in the presence of PAHs. The mechanism was the sorption of pollutants by biochar. It also supports the growth of microorganisms through its porous structure and because biochar itself is a nutrient carrier for microorganisms. It also improves soil water storage capacity and air circulation (Li et al. 2019).

### Polysterene

Bacteria of the genus *Pseudomonas* sp. and *Acinetobacter johnsonii* Bouvet and Grimont were used in the biodegradation of polystyrene (PS). This microflora has the properties of PS modification by introducing hydroxyl groups and new chemical bonds. As a result, a greater degree of hydrophilicity of the PS surface was achieved. The role of the enzyme alkane-1-monooxygenase (AlkB) in the biodegradation (hydroxylation) of PS was confirmed. This enzyme has been identified in these microorganisms (Kim et al. 2021).

### Mycrocystins

Microcystins are compounds produced by cyanobacteria in surface waters. These chemicals enter the soil environment along with irrigation water, where they pose a toxic effect. Microcystins are absorbed from contaminated soil by plants and bioaccumulated in their tissues, e.g., in vegetables such as lettuce, cucumber, or carrot. The consumption of contaminated food can lead to human exposure to these toxic compounds that can damage the kidney or liver (Cao et al. 2022).

### Metals

#### Cd

In a study by Viji et al. (2021), bacteria were examined in collected soil samples, contaminated with cadmium. Four different strains of bacteria were selected (VITATJM1, VITATJM2, VITATJM3, and VITATJM4). PGPR tests were performed, evaluating the production of, among others, indole-3-acetic acid, hydrogen cyanide, and ammonia and phosphate dissolution. The bacterial cultures VITATJM3 and VITATJM4 have proved to be particularly effective in this regard. The plant *Pennisetum purpureum* Schumach was evaluated in the pot studies. In their conclusions, the authors emphasized that *P. purpureum*–assisted remediation may be effective in removing cadmium from the soil (Viji et al. 2021).

#### Pb

The paper by Kushwaha et al. (2018) reviews the presence of lead in the soil-microorganism-plant system and methods of bioremediation of soils that are contaminated with this element. Pb bioavailability (and hence toxicity) is determined by its speciation. Living organisms have developed by evolution detoxification mechanisms that are useful in bioremediation processes (Kushwaha et al. 2018). The following factors influence the speciation of Pb in soil and its mobility: pH, soil composition (iron oxides, clay materials, organic colloids, organic matter) (Kushwaha et al. 2018).

The bioaccumulation of HMs by plants affects the rhizosphere of plants, which can be assisted by phytoremediation. The aim of the paper by Zhang et al. 2022 was to identify the mechanisms of lead accumulation by assessing the effect of nitrilotriacetic acid (NTA) on the growth of *Athyrium wardii* (Hook.) Makino. Following the NTA application, it was discovered that the accumulation of Pb increased (14.3%) and the activity of the microorganisms decreased slightly. The above influences changes in the soil of the rhizosphere: a decrease in pH (0.37 units), an increase in the content of dissolved organic carbon (7.6%), and an enhancement of urease, catalase, and phosphatase activity. The results, among others, emphasized that the application of NTA may stimulate microflora to sustain the activity in the root zone in soils with a high lead level (Zhang et al. 2022).

#### Pb, Cd, Zn

The removal of metal(loids) from the soil can be performed using in situ remediation (phytoextraction). In this technique, (hyper)accumulation plant species can be used. In the original work of Yu et al. (2022), field studies were carried out using (hyper)accumulatory species to assess the impact of rhizosphere on contaminated soils and to analyze the variability of the diversity and microorganisms of the rhizosphere community (Mapelli et al. 2022). The field trials lasted 2 years. The results emphasized that two species—*Xanthium strumarium* L. and *Bidens pilosa* L.—showed a higher ability to phytoextract metal ions. In the root zone of all plants, a reduced level of Pb, Cd, and Zn (due to the synthesis of DTPA in the root zone) was found. The authors emphasized that the cultivation of the assessed plants helps reconstruct the communities of bacteria (*Proteobacteria* sp., *Actinobacteriota* sp., *Chloroflexi* sp., and *Acidobacteriota* sp.) and fungi (*Ascomycota* sp., *Basidiomycota* sp., and *Mortierellomycota* sp.) in the soil, which can show a beneficial influence on the phytoextractive capacity of (hyper)accumulators (Yu et al. 2022).

#### Cd, Zn

Sedum plants are among the Cd/Zn phytoextractors. These plants show great potential to improve the effectiveness of phytoremediation (use of rhizospheric/endophytic microbiome). Two plants were evaluated in field trials: non-hyperaccumulating *Sedum alfredii Hance* and hyperaccumulating *Sedum plumbizincicola* X.H.Guo & S.B.Zhou ex L.H.Wu (*S. alfredii* and *S. plumbizincicola*) in terms of the ecotype of the bacterial microbiome. The potential for both the transport of heavy metal ions and their uptake by sedum was also assessed. In the results obtained, the authors emphasized that the hyperaccumulating ecotype was effective in phytoremediation, both in cultivated fields and in mining areas. The sedum rhizosphere was dominated by *Proteobacteria*, *Actinobacteria*, and *Acidobacteria*. In the conclusions, it was underlined that the taxa related to the hyperaccumulation of Zn and Cd were similar. Nine types of bacteria showed a positive correlation with hyperaccumulation of Cd/Zn (Wu et al. 2022).

#### Cd, Zn, Pb, Cu

Bacterial-assisted phytoextraction is increasingly being applied in the remediation of soils contaminated with heavy metals. It has not been confirmed whether phytoextraction is facilitated by abscisic acid (ABA) in plants by microorganisms. For this purpose, selected plants were inoculated with ABA-catabolizing bacteria (*Rhodococcus qingshengii* Xu). The results showed that in *S. alfredii*, *Chrysopogon zizanioides* (L.) Roberty, *Lolium perenne* Host L., *Brassica juncea* (L. Czern.), and *Solanum nigrum* L., the concentration of Cd, Zn, Pb, and Cu increased by 28.8–331.3%, 8.5–393.4%, 21.2–222.5%, 14.7–115.5%, and 28.3–174.2% (compared to the control). On the basis of the assessment of the potential for phytoremediation, it was concluded that the bacteria significantly increased the efficiency of phytoextraction from the soil. In their conclusions, the authors stressed that there was a significant correlation between the influence of microorganisms on the concentration of Zn and Cd in plants with the metabolism of ABA. There was no such correlation in the case of Pb and Cu (Wang et al. 2022).

### Metalloids

#### As

It is possible to transform toxic metalloids into less toxic chemical forms. This applies, inter alia, to arsenic (As), its concentration and form that determines its toxicity to plants. In the original work by Tripathi et al. (2017), the influence of *Trichoderma* sp. on the regulation of arsenic toxic effects in chickpea plants was evaluated. The plant (*Cicer arietinum* L.) was grown in soil and inoculated with two *Trichoderma* strains: PPLF-28 sensitive and M-35 tolerant. A comparable level was found in plants with different strains. However, the concentration of inorganic and organic arsenic compounds (iAs) differed. It also turned out to be different. According to the gene evaluation, the tolerant strain contributed to the increased coping potential of plants with As stress (Tripathi et al. 2017).

The paper by Prasetya et al. (2023) provides a comprehensive review of metal–organic frameworks (MOFs) for the adsorptive removal of pharmaceutically active compounds (PhACs) from water and wastewater. A valuable information on the potential use of MOFs for the removal of persistent and prevalent emerging contaminants, such as PhACs, from water and wastewater. These findings can also be relevant to the development of more effective remediation methods for PAHs in soil (Prasetya et al. 2023).

PhACs are among the most persistent and prevalent emerging contaminants in the environment. The conventional treatment methods, such as activated carbon adsorption, have limitations in removing PhACs due to their complex chemical structures and low concentrations. This limitation is also relevant to PAHs, which are among the most persistent organic pollutants found in soil. PAHs are primarily formed during incomplete combustion of organic matter, such as fossil fuels and biomass, and can persist in the environment for decades. They are toxic to organisms and can cause soil and water contamination, and some are carcinogenic.

Recent studies have investigated the use of MOFs for the adsorptive removal of PAHs from soil and water. For example, a study (Guo et al. 2021) reported that the MOF MIL-101(Cr) was effective in removing PAHs from soil, with a maximum adsorption capacity of 316 mg/g for phenanthrene. Another study by Chen et al. (2021) reported the successful use of MOF-808 for the removal of PAHs from water.

## Future perspectives

Rhizoremediation is believed to overcome the disadvantages of conventional phytoremediation by taking advantage of benefits from the capacity of soil microorganisms to support the phytoremediation process. However, there are no practical application examples. The problem of scientific research is the high variability of experimental factors, which makes it difficult to draw unambiguous conclusions: uneven distribution of pollutant concentrations, coexistence of various pollutants, and low bioavailability of pollutants that are adsorbed on soil particles. There is a lack of real-scale field research on rhizoremediation supported with functional materials. The strategy is to create a library of strains capable of degrading pollutants in combination with plant species for which they represent symbiotes and with soil amendments (Ben-Othman et al. 2020). The mechanism in the plant is triggered in response to contamination with toxic elements: glutathione, phytochelatins, amino acids (e.g., histidine), phytin, organic acids, ion-binding proteins, and flavonoids.

The benefits of rhizoremediation include low cost and high efficiency (full remediation of contaminated areas is possible). This is an environmentally friendly method that can be used in large areas, is easy to carry out, and has favorable operating costs. On the other hand, the disadvantages are as follows: high dependence on the effective association of a plant species with symbiotes, takes time, not all pollutants can be removed in this way, it is possible to cause a toxic effect, and seasonality related to the vegetation season of plants.

## Conclusions

The goal of this paper was to provide a comprehensive overview of rhizoremediation supported with functional materials as a promising soil remediation technique. The review highlights the potential synergistic effects of the triad of microorganisms, plants, and functional materials and the role of each component in reducing the toxicity of pollutants and improving remediation efficiency.

Based on the review of the literature, it is evident that rhizoremediation supported with functional materials is a relatively new and exciting area of research. However, there is still a lack of field-test experiments, even though a multitude of laboratory works have been reported. The findings of this study have several implications for future research to consider the potential effects of using a triad plant-microorganism-soil amendment on the remediation efficiency of soils in real systems contaminated with several pollutants.

While much bioremediation research has focused on phytoremediation, bioremediation, and functional soil amendments, rhizoremediation supported with functional materials has recently attracted considerable attention. Early works in this area focused primarily on the investigation of mechanisms and the role of each player in this three-component system. Few researchers have addressed the problem of conducting the process at the higher TRL level.

The limitations of this technique lie in the lack of practical protocols from real contaminated field studies, which leaves many questions unanswered. Modeling the rate and efficiency of these remediation methods and conducting field-scale experiments are crucial to determine the real potential and effectiveness of this remediation technique.

This study has highlighted the significance of rhizoremediation supported by functional materials in soil remediation. The synergistic effect of the triad, namely microorganisms, plants, and functional materials, was found to be more effective than traditional remediation methods. The use of this technique has the potential to reduce soil contamination with toxic chemicals more effectively and in an environmentally friendly manner. However, the lack of field-test experiments remains a limitation, and further research is required to evaluate the effectiveness of rhizoremediation in real contaminated systems. To provide a more detailed understanding of the application of rhizoremediation with functional materials, examples of the key elements presented are necessary. For instance, the combination of titanium nanodioxide, silicon nanoparticles, and carbon nanotubes has been found to improve the effectiveness of rhizoremediation for different types of soil contaminants. Additionally, the use of specific plant species and endophytic microorganisms has been shown to enhance the efficiency of rhizoremediation in specific contaminated sites. The role of functional materials, including their capacity to adsorb pollutants and provide a habitat for the growth of rhizospheric microflora, is crucial in the success of the process. Therefore, the integration of these key elements can lead to more effective and sustainable soil remediation practices.

This paper has highlighted the potential of rhizoremediation as an effective and eco-friendly technique for soil remediation. However, despite numerous laboratory studies, there is still a significant lack of field-test experiments. Only a few studies have examined the efficacy of rhizoremediation under field conditions. Therefore, there is a need for more field studies to validate the efficiency of this technique for soil remediation.

According to some studies, the use of a combination of plants, microorganisms, and functional materials can significantly enhance the remediation efficiency of soils contaminated with multiple pollutants. For example, one study found that the addition of chelating agents (such as ethylenediaminetetraacetic acid, EDTA) to the soil improved the phytoextraction efficiency of heavy metals by up to 50%. Another study demonstrated that the use of a microbial consortium containing *Rhodococcus qingshengii* and plant species such as *Lolium perenne* and *Sedum alfredii* resulted in a 28.8 to 331.3% increase in Pb, Cd, Cu, and Zn levels in contaminated soil.

Some studies have also reported a synergistic effect among plants, microorganisms, and functional materials, indicating that each component plays a unique complementary role in the remediation process. For instance, the presence of mycorrhizal fungi in the rhizosphere of plants can enhance the nutrient uptake and growth of the host plants. The addition of biochar to the soil can improve its physical, chemical, and biological properties, leading to enhanced plant growth and pollutant removal.

Our findings suggest that the use of a triad plant-microorganism-soil amendment can lead to a more efficient and effective remediation process. This is supported by studies that have reported a synergistic effect between different components of the triad. For example, the use of plant–microbe-fungus combinations has been shown to be effective in removing heavy metals from contaminated soil. The efficiency of rhizoremediation can be improved by optimizing the use of functional materials such as biochar, chelating agents, and microbial consortia.

## Data Availability

Not applicable.
